# A deep learning model to detect pancreatic ductal adenocarcinoma on endoscopic ultrasound-guided fine-needle biopsy

**DOI:** 10.1038/s41598-021-87748-0

**Published:** 2021-04-19

**Authors:** Yoshiki Naito, Masayuki Tsuneki, Noriyoshi Fukushima, Yutaka Koga, Michiyo Higashi, Kenji Notohara, Shinichi Aishima, Nobuyuki Ohike, Takuma Tajiri, Hiroshi Yamaguchi, Yuki Fukumura, Motohiro Kojima, Kenichi Hirabayashi, Yoshihiro Hamada, Tomoko Norose, Keita Kai, Yuko Omori, Aoi Sukeda, Hirotsugu Noguchi, Kaori Uchino, Junya Itakura, Yoshinobu Okabe, Yuichi Yamada, Jun Akiba, Fahdi Kanavati, Yoshinao Oda, Toru Furukawa, Hirohisa Yano

**Affiliations:** 1 Department of Pathology, Kurume University School of Medicine, Kurume, 830-0011 Japan; 2 Department of Diagnostic Pathology, Kurume University Hospital, Kurume, 830-0011 Japan; 3 Medmain Research, Medmain Inc, Fukuoka, 810-0042 Japan; 4 Department of Pathology, Jichi Medical University, Shimotsuke, Tochigi 329-0498 Japan; 5 Department of Anatomic Pathology, Graduate School of Medical Sciences, Kyushu University, Fukuoka, 812-8582 Japan; 6 Department of Pathology, Research Field in Medicine and Health Sciences, Medical and Dental Sciences Area, Research and Education Assembly, Kagoshima University, Sakuragoaka, 890-8544 Japan; 7 Department of Anatomic Pathology, Kurashiki Central Hospital, Kurashiki, 710-8602 Japan; 8 Department of Pathology and Microbiology, Saga University, Saga, 849-8501 Japan; 9 Department of Pathology, Saga University Hospital, Saga, 849-8501 Japan; 10 Department of Pathology, Shizuoka Cancer Center, Shizuoka, 411-8777 Japan; 11 Department of Pathology, Tokai University Hachioji Hospital, Tokyo, 192-0032 Japan; 12 Department of Pathology, Saitama Medical University, Saitama, 350-0495 Japan; 13 Department of Human Pathology, School of Medicine, Juntendo University, Tokyo, 113-8421 Japan; 14 Division of Pathology, Exploratory Oncology Research and Clinical Trial Center, National Cancer Center, Kashiwa, 277-8577 Japan; 15 Department of Pathology, Tokai University School of Medicine, Isehara, 259-1193 Japan; 16 Department of Pathology, Faculty of Medicine, Fukuoka University, Fukuoka, 814-0180 Japan; 17 Department of Investigative Pathology, Tohoku University Graduate School of Medicine, Sendai, 980-8575 Japan; 18 Department of Anatomic Pathology, Tokyo Medical University, Tokyo, 160-0023 Japan; 19 Division of Gastroenterology, Department of Medicine, Kurume University School of Medicine, Kurume, 830-0011 Japan

**Keywords:** Gastroenterology, Medical research

## Abstract

Histopathological diagnosis of pancreatic ductal adenocarcinoma (PDAC) on endoscopic ultrasonography-guided fine-needle biopsy (EUS-FNB) specimens has become the mainstay of preoperative pathological diagnosis. However, on EUS-FNB specimens, accurate histopathological evaluation is difficult due to low specimen volume with isolated cancer cells and high contamination of blood, inflammatory and digestive tract cells. In this study, we performed annotations for training sets by expert pancreatic pathologists and trained a deep learning model to assess PDAC on EUS-FNB of the pancreas in histopathological whole-slide images. We obtained a high receiver operator curve area under the curve of 0.984, accuracy of 0.9417, sensitivity of 0.9302 and specificity of 0.9706. Our model was able to accurately detect difficult cases of isolated and low volume cancer cells. If adopted as a supportive system in routine diagnosis of pancreatic EUS-FNB specimens, our model has the potential to aid pathologists diagnose difficult cases.

## Introduction

Pancreatic ductal adenocarcinoma (PDAC) is a disease with a poor prognosis among gastrointestinal cancers^1,2^. Although long-term survival rates remain poor, surgical resection is the mainstay of treatment for PDAC^3^. Poor prognosis is due to the fact that the PDCA is already advanced at the initial diagnosis and that effective treatment methods have not been developed^4^. However, in recent years, the diagnostic results of endoscopic ultrasonography-guided fine-needle aspiration cytology (EUS-FNA) and endoscopic ultrasonography-guided fine-needle biopsy (EUS-FNB) for PDAC have improved and have had a positive impact on the diagnostic and therapeutic strategy of PDAC.

In the past, trans-papillary pancreatic juice cytology was the mainstay for preoperative diagnosis of pancreatic cancer^5,6^. It was difficult to obtain adequate specimens for ERCP (endoscopic retrograde cholangiopancreatography) because of the collection of cells from thin pancreatic ducts. EUS-FNA and EUS-FNB changed this and made it easier to obtain adequate specimens via direct punctures leading to improvements in diagnostic results. EUS-FNA can benefit from the addition of rapid on-site evaluation (ROSE) which provides immediate feedback, and it has improved diagnosis. However, there are still issues such as the limited number of facilities where ROSE can be performed. On the other hand, more recently, EUS-FNB is being used more than EUS-FNA for tissue acquisition^7^ as it has been reported to provide stable diagnostic results via improvements to the puncture needle^8–10^.

**Figure 1:**
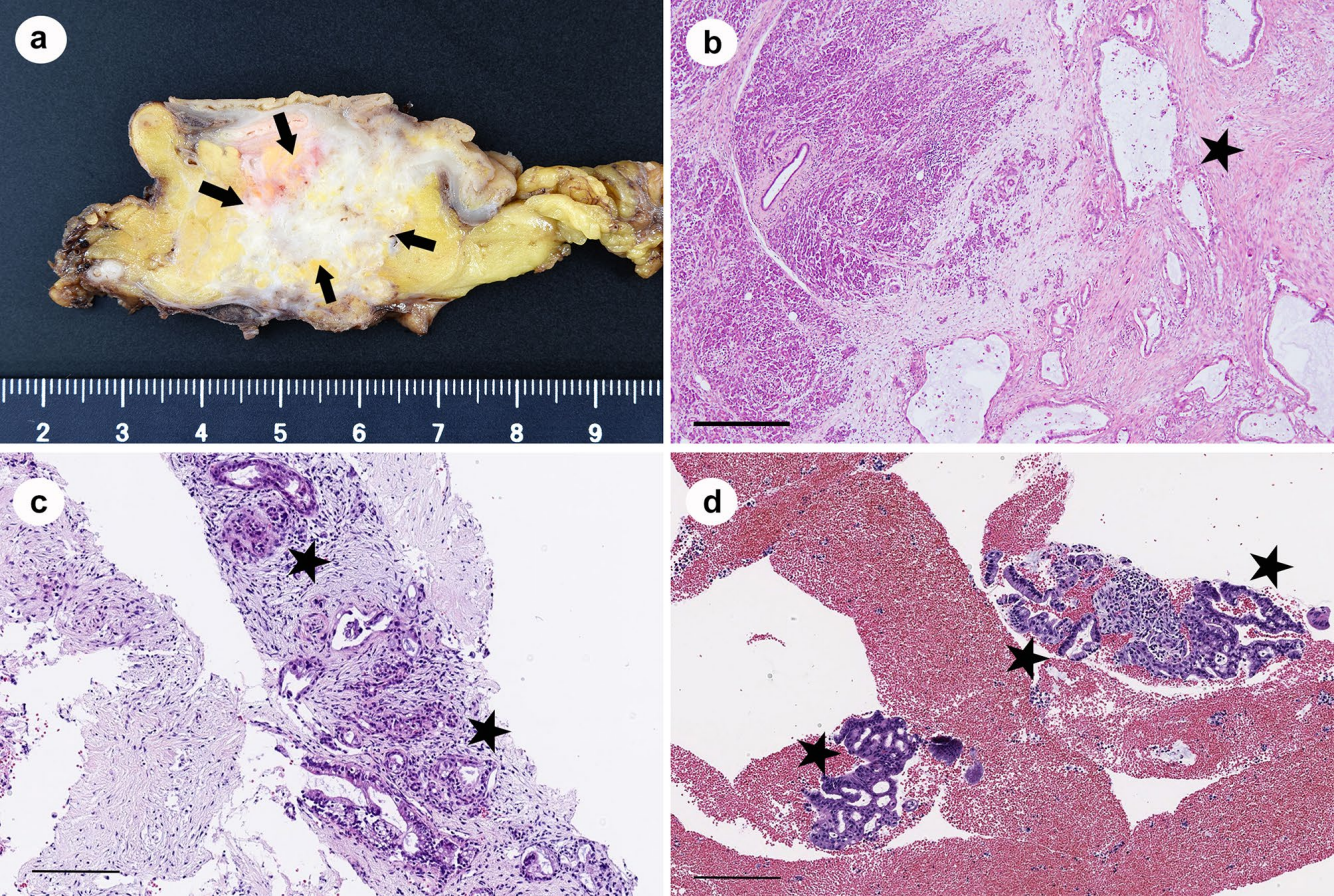
Histology of pancreatic ductal adenocarcinoma (PDAC). A representative resected PDAC in white color and showed an invasive growth pattern (**a**) Histologically, tumor cells with distinct glandular formation were infiltrating and proliferating within abundant stroma (**b**) The representative EUS-FNB specimen consists of the IDC (**c**) and ICC (**d**) foci within the blood or fibrin component. Arrows (**a**): tumor; stars (**b**–**d**): pancreatic ductal adenocarcinoma. Scale bars: 500 μm (**b**), 200 μm (**c**, **d**).

Clinically, diagnosis of pancreatic tumors has improved, but pathological diagnosis remains difficult. The reason is that the amount of tissue collected is small and fragmented. The majority of pancreatic cancer histologies are adenocarcinomas (Fig. 1a,b). The cancer cells in EUS-FNB tissue are mainly invasive ductal carcinoma components (IDC) (Fig. 1c) and fragmented isolated carcinoma components (ICC) (Fig. 1d). IDC typically show adenocarcinomas in desmoplastic stroma. The desmoplastic stroma is a reliable diagnostic clue because it is a result of stromal invasion by adenocarcinoma. On the other hand, ICCs are fragmented cancer cells contained within blood cells, which is often difficult to diagnose because only cellular atypia is available. EUS-FNB tissues often contain ICCs in greater abundance than IDCs. General pathologists find it difficult to diagnose such specimens; even pathologists who specialize in pancreatic pathology cannot easily diagnose pancreatic pathology based on ICC alone. Therefore, to obtain good diagnostic results, pathologists would need specimens that contain a variety of tissue components in addition to ICCs.

Artificial intelligence based on a deep learning model that can assist the pathologists in evaluation of such difficult cases for diagnosis may be of great help. Deep learning models, especially convolutional neural networks (CNNs), have found numerous successful applications in the computational pathology^11–23^. One of the primary applications in histopathology is performing automatic cancer detection in whole-slide images (WSIs)^22,23^. However, as far as we are aware, there has not been any previous applications of deep learning to detect adenocarcinoma on pancreatic EUS-FNB specimens.

In this paper, we propose a deep learning model for pancreatic EUS-FNB WSI classification. We used a combination of transfer learning and fully-supervised learning to train an EfficientNet-B1^22,23^ CNN on a dataset consisting of 372 WSIs. We then evaluated the model on a test set of 120 WSIs with a pathological diagnosis matched by three pancreatic pathologists, achieving high ROC AUC performance.

## Results

### A deep learning model for PDAC classification of EUS-FNB samples

**Figure 2:**
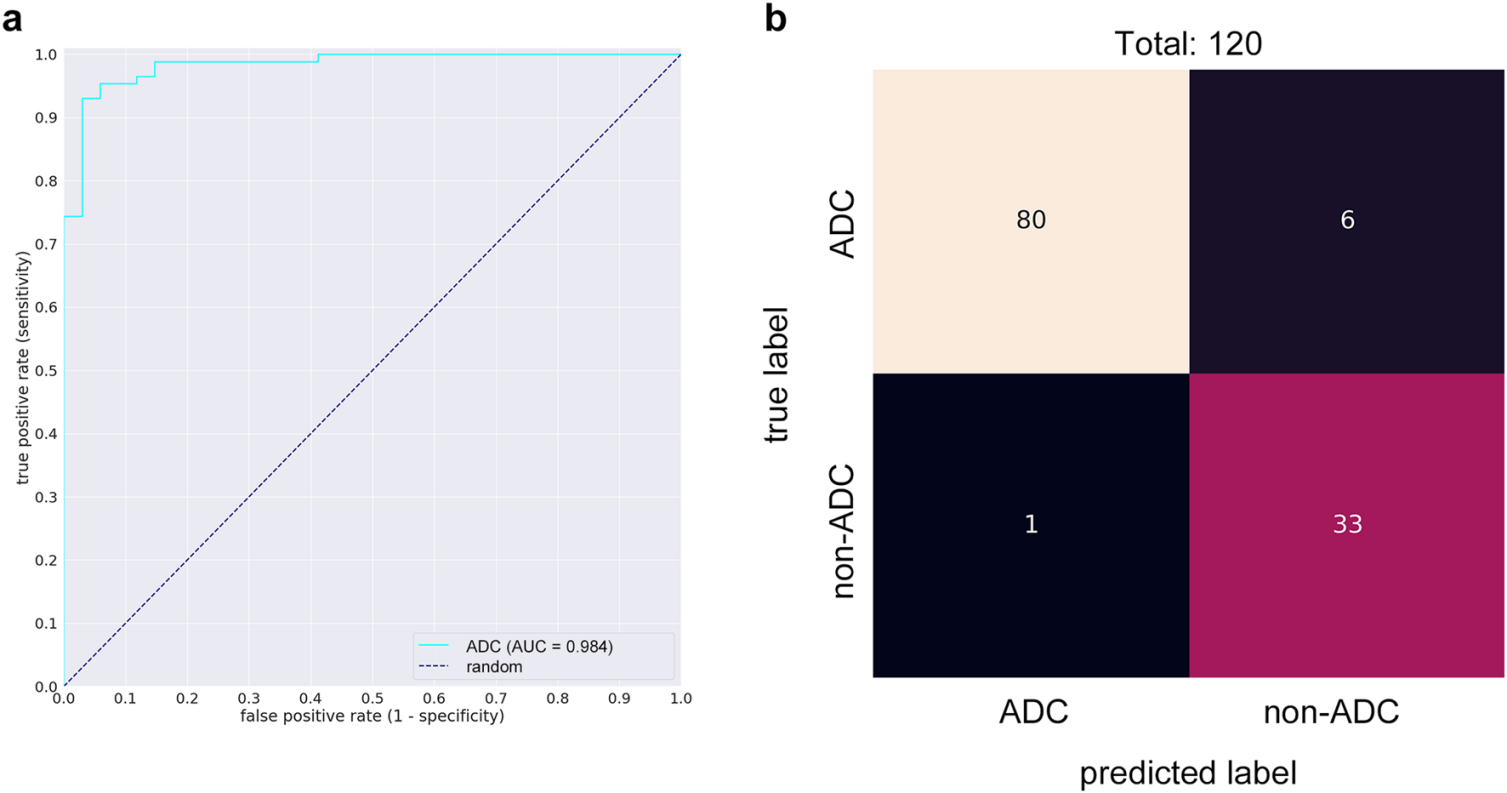
Evaluation performance of the model on the test set. The ROC curve of ADC WSI classification using a test set of 120 verified WSIs (**a**). Confusion matrix for WSI binary classification into ADC and non-ADC on the test set (**b**).

The aim of this study was to train a deep learning model to evaluate PDAC in EUS-FNB WSIs. The final dataset used for developing the model consisted of 532 WSIs from Kurume University. The dataset was divided as follows: a training set of 372 WSIs (161 PDAC), a validation set of 40 WSIs (20 PDAC), and a test set of 120 (86 PDAC) (see Supplementary Fig. S1 online). The test set was derived from completely agreed WSIs of independent reviews of 182 WSIs by three pancreatic pathologists (Y.N., N.F and T.F). (see Supplementary Table S1 online). 62 WSIs that had a disagreement on the diagnosis were considered as “indeterminate” and were excluded from the test set. Fleiss's Kappa value, which assesses agreement with the diagnosis, was 0.677, which was determined to be substantial agreement. We evaluated the model on the test set and computed a combination of metrics (Table 1). The model has high Receiver Operator Curve (ROC) area under the curve (AUC) (0.9836; CI [0.9603–0.9977]), accuracy (0.9417; CI [0.8917–0.975]), f1-score (0.9581; CI [0.915–0.9827]), sensitivity (0.9302; CI [0.8602–0.9753]) and specificity (0.9706; CI [0.9091–1]). Figure 2 shows the ROC curve (Fig. 2a) and confusion matrix (Fig. 2b).

**Table 1:** A variety of metrics computed on the test sets. A threshold of 0.47 was used.

Metrics	Value	Confidence interval
ROC AUC	0.9836	[0.9603–0.9977]
Log loss	0.3419	[0.2949–0.3864]
Accuracy	0.9417	[0.8917–0.975]
MCC	0.8667	[0.7622–0.9473]
f1-score	0.9581	[0.915–0.9827]
Sensitivity (TPR)	0.9302	[0.8602–0.9753]
Specificity (TNR)	0.9706	[0.9091–1]
Precision (PPV)	0.9877	[0.9571–1]
Negative predictive value (NPV)	0.8462	[0.7297–0.9512]
False discovery rate (FDR)	0.0123	[0–0.0429]

**Figure 3:**
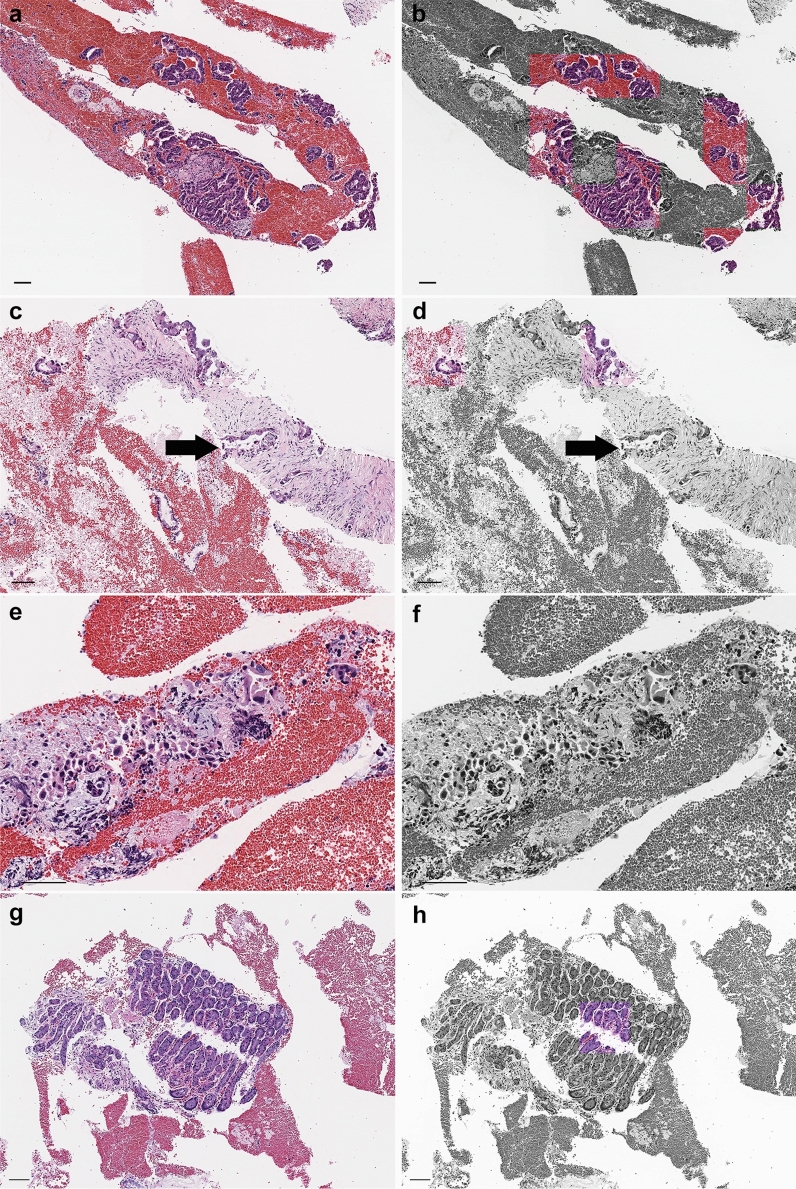
Examples of model prediction outputs for pancreatic ductal adenocarcinoma (PDAC). EUS-FNB samples are mainly composed of fragmented tissues. Our model was able to detect cancer cells selectively among the isolated cells in the specimen (**a**, **b**). However, the identification of invasive cancer cells (arrow) was not always made accurately (**c**, **d**). The detection of cancer cells in small cluster areas was difficult (**e**, **f**). In the false positive case, gastric glands were mislabelled as adenocarcinoma (**g**, **h**). Scale bars: 100 μm.

**Figure 4:**
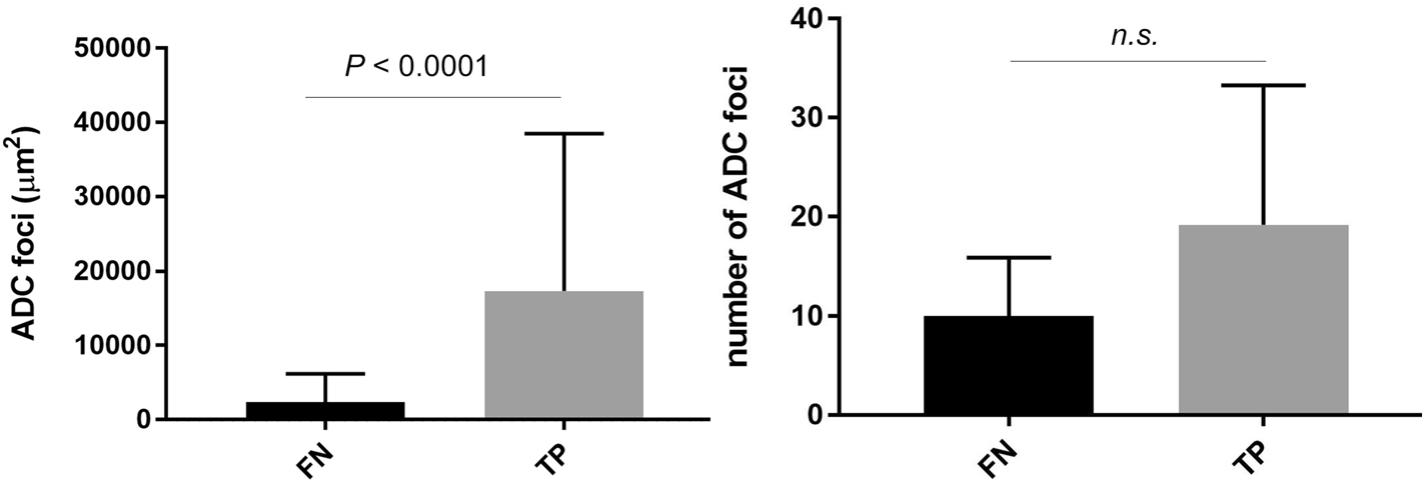
Effect of area and number of pancreatic ductal adenocarcinoma (PDAC) foci on false-negatives. The area of ADC foci was significantly larger in the true positives than in the false negatives (left panel). On the other hand, there was no significant difference in the number of ADC foci between the true positives and false negatives (right panel).

In true positives, cancer cells were accurately detected and no background blood cells or contamination were detected (Fig. 3a,b). Interestingly, despite the inclusion of IDC, some of the IDC nests were not recognized in the true positives (Fig. 3c,d). Moreover, in false negatives, small cancer nests of ICC were not detected (Fig. 3e,f). The area of cancer cell foci was predominantly larger in the true positives than the false negatives (Fig. 4). On the other hand, there was no significant difference in the number of cancer cell foci between the true positives and false negatives (Fig. 4). There was a false positive case which had mislabelled contaminated gastric gland tissues (Fig. 3g,h).

## Discussion

In the present study, our deep learning model established using pancreatic EUS-FNB specimens containing fragmented pancreatic tissue and large amounts of blood was shown to have high accuracy (0.9417; CI [0.8917–0.975]), sensitivity (0.9302; CI [0.8602–0.9753]) and specificity (0.9706; CI [0.9091–1]). It is particularly noteworthy that it was possible to extract even ICCs, which are difficult for pathologists to evaluate. Although there were false negatives, the results were dependent on the area of PDAC foci, not on the sample volume. Our deep learning model can be effectively used as a diagnostic support system for EUS-FNB specimens with a large amount of blood and fibrin.

The model was trained on a small dataset of 372 WSIs and evaluated on a test set of 120. The model achieved high ROC AUC performance of 0.984 (CI: [0.9603–0.9977]), which is comparable to the performance of classification models for other malignant tumors^21–24^. The maximal use of the training set, which consisted in the annotation by the expert pancreatic pathologists of all the adenocarcinoma cells within a specimen (ICCs as well as IDC components), in combination with the adopted training methodology (transfer learning and fully-supervised learning) has been an important factor in achieving the high performance.

The trained model had a few false-negative and false-positive predictions on the test set. In some false-negative cases, the foci were significantly smaller than that of true-positive foci. This is most likely due to the facts that there were only a limited number of cases within the training set that had focus areas with cancer infiltration and stromal induction. This is in contrast to colonic, gastric, and prostate cancer biopsy specimens where there are a large number of cases with such findings. Interestingly, even in true-positive WSI diagnosis cases, some of the IDC infiltrations were not detected, which are representative of PDAC findings. This could be due to the limited number of annotations of IDC infiltrations, which means that our model might not have learned from enough examples to be able to detect all instances of IDC. As for the false positives, our deep learning model detected contaminated tissue (gastric glands) as cancer cells. The contamination was due to the process of fine-needle puncture that went through the gastric glands before reaching the targeted pancreas. The false positive was most likely due to the limited number of contaminated tissue fragments within the training set. False positives are less of a concern than false negatives in practical diagnostic workflows given that the WSI diagnosis would always be revised and confirmed by a pathologist who has studied general pathology.

Despite the high performance of the model, there are still a few limitations. One limitation of our model is that the training and test WSIs were all obtained from a single institution, and, therefore, it is uncertain how well the model would perform on WSIs obtained from a different institution. Another limitation is that the test set size is small (n = 120), and it might not include all the potential variations of cases that could be encountered; therefore, it is difficult to obtain a good approximation of the true performance of the generalization of the model. However, given that the incidence of pancreatic cancer is rare as compared to gastric and colonic cancers^25^, far fewer biopsies are being performed, making it difficult to obtain a large WSI dataset from a single institution.

As future work, we intend to further develop and evaluate our model on multiple test sets obtained from different medical institutions in order to assess its generalization performance and move closer towards the adoption of such assistive models in routine histopathological diagnoses workflows.

## Methods

### WSIs from patients with pancreatic disease

A total of 594 WSIs who underwent EUS-FNB at Kurume University Hospital (Kurume, Japan) between January 2010 and March 2020 were enrolled in this retrospective study. WSIs that had a special subtype of PDAC and metastatic tumor were excluded from this study. Informed consent to use histopathological samples and pathological diagnostic reports for any present or future research studies had previously been obtained from all patients prior to the surgical procedures and the patients were aware that at any time they could change their mind and opt-out from ongoing research studies by going on the Kurume University official website. This study was approved by the Research Ethics Committee of Kurume University (#19182) on November 18, 2019, which conforms to the guidelines of the Declaration of Helsinki.

The tissue specimens were obtained from patients with pancreatic tumors referred to Kurume University Hospital who were determined to be eligible for EUS-FNB. EUS-FNB specimen collection was performed mainly using a 22G/25G puncture needle; 15–20 strokes (average: 2.7 strokes) and 3–5 sessions were performed. The specimen was fixed in neutral buffered formalin solution. Rapid on-site evaluation was also performed in each case. For each WSIs a primary pancreatic lesion hematoxylin & eosin (HE) stained histopathological specimen was collected after histopathological review by surgical pathologists and scanned into a WSI at a magnification of 20×. Pathological diagnosis was performed according to the 2019 World Health Organization Classification of Tumors of the Digestive system tumors^26^. Basically, the tissues obtained by EUS-FNB were found to be a mixture of distinct and fragmented pancreatic tissue on a background of various degrees of blood. For this study, we defined the tubular adenocarcinoma found in clear pancreatic tissue as IDC and the fragmented cancer cells as ICC. IDC was defined as adenocarcinoma with preserved morphology as an invasive ductal carcinoma of the pancreatic parenchyma. On the other hand, ICC was defined as adenocarcinoma with no association with the pancreatic parenchyma and indistinct morphology as an invasive ductal carcinoma.

### Datasets and annotations

The dataset obtained from Kurume University consisted of 594 WSIs, of which 182 WSIs from December 2019 and 2020 April were selected as test sets of which 62 WSIs were excluded due to disagreements on their diagnoses by a set of three expert pancreatic pathologists. The dataset was solely composed of pancreatic EUS-FNB WSIs. 412 WSIs were used for annotation and were looked over carefully and verified by two independent pathologists prior to annotation. The WSIs were manually annotated by a group of 18 pancreatic surgical pathologists (specialists) who perform routine pancreatic EUS-FNB histopathological diagnoses by drawing around the areas that corresponded to one of the eleven labels (Table 2). Annotations performed by pathologists were modified (if necessary), confirmed, and verified by another pathologist (see Supplementary Fig. S2 online). The resulting WSIs contained multiple annotation labels; however, given that the goal was to train a binary classification model, a diagnosis WSI label of adenocarcinoma (ADC) or non-ADC was also assigned to the WSI based on the presence of PDAC annotations. The types of annotation labels, the number of annotations for each label and the annotation labels corresponding to the binary classification are summarized in Table 2.

**Table 2:** Summary of annotation labels and model output labels.

Annotation label	Label description	Number of verified annotations	AI output label
Pancreatic duct	Normal duct	872	Non-ADC
Acinus	Normal acinus	454	Non-ADC
Islets of Langerhans	Normal islet cell	10	Non-ADC
ADC	Adenocarcinoma	7250	ADC
NET	Neuroendocrine tumor	695	Non-ADC
SPN	Solid pseudopapillary neoplasm	0	Non-ADC
ACC	Acinar cell carcinoma	47	Non-ADC
No tumor	Pancreatitis	515	Non-ADC
AIP	Autoimmune pancreatitis	11	Non-ADC
Background	Hemocyte, epithelial contamination, fibrin, non-neoplastic stroma	6207	Non-ADC
Indeterminate cell	Cells impossible to distinguish	7858	Non-ADC

### Deep learning models

For the current study we used the EfficientNet-B1^27^ architecture, which is a smaller version of the state-of-the-art EfficientNet architecture that has achieved a good compromise in performance and model size. We trained the model using transfer learning and fully-supervised learning. The model was instantiated by using the fully-convolutional layers of an EfficientNetB1 CNN that pre-trained on ImageNet and appending a global average pooling layer followed by a fully-connected classification layer with a single sigmoid output. The WSIs were down-sampled to a magnification of 10 × from 20 × without loss of classification performance. The large size of the WSIs, typically in the tens of thousands of pixels along each dimension, poses a computational challenge, making it difficult to apply a CNN to the entire WSI at once. We followed the typical approach of breaking down the WSIs into thousands of smaller fixed-sized tiles and applying the CNN on the tiles, rather than directly on WSIs. The training WSIs were divided into overlapping fixed-sized tiles of 512 × 512 pixels with a stride of 256 pixels. During training, the tiles were fed into the model using balanced batch sampling with real-time data augmentation consisting of variations in brightness, saturation, and rotation.

The fully-supervised training method that we used is similar to the fully-supervised method described in^22^. The model was trained for a total of 50 epochs and the model’s performance was monitored on a validation set. We used early stopping, where we selected the model from the epoch with the lowest validation loss. To obtain a WSI classification, the model was applied with a stride of 256 pixels in a sliding window fashion resulting in a probability output for ADC for each tile. We then took the maximum probability as the probability for the WSI. If this probability was greater than a threshold (0.5) then the WSI is predicted as ADC, non-ADC otherwise. This means that a WSI is assigned the diagnosis of ADC if at least one tile was predicted as ADC.

### Software and statistical analysis

The Fleiss' kappa statistics were performed to assess the pathological diagnostic concordance of three pancreatic pathologists for selecting appropriate test sets. Fleiss' kappa is a measure of inter-rater agreement used to determine the level of agreement between two or more raters when the method of assessment, known as the response variable, is measured on a categorical scale^28^. The Kappa values were calculated using Microsoft Excel 2016 MSO (16.0.13029.20232) 64 bit and interpreted as follows: < 0.0, poor agreement; 0.01–0.20, slight agreement; 0.21–0.40, fair agreement; 0.41–0.60, moderate agreement; 0.61–0.80, substantial agreement; 0.81–1.00, almost perfect agreement.

We used the TensorFlow framework to implement the deep learning models, the scikit-learn package was used to calculate the metrics and matplotlib was used to plot the ROC curves. We used the bootstrap method with 1000 iterations to estimate the 95% CIs of the AUCs. The number and area of cancer cell foci were calculated using ImageJ (https://imagej.nih.gov/ij/) software in all false-positives (6 WSIs) and randomly selected true-positives (6 WSIs).

## Supplementary Information

Supplementary Information

## Data Availability

Due to specific institutional requirements governing privacy protection, all of the datasets used in this study are not publicly available.
