# Supplementary material for: A deep learning model to detect pancreatic ductal adenocarcinoma on endoscopic ultrasound-guided fine-needle biopsy
Source: Sci Rep. 2021 Apr 19;11:8454. doi: 10.1038/s41598-021-87748-0 (PMC8055968; doi:10.1038/s41598-021-87748-0)
Supplement: Supplementary file 1 — Supplementary Information [file 41598_2021_87748_MOESM1_ESM.pdf]

A deep learning model to detect pancreatic ductal adenocarcinoma on endoscopic ultrasound-guided fine-needle biopsy

Yoshiki Naito[1,2*], Masayuki Tsuneki[3*], Noriyoshi Fukushima[4], Yutaka Koga[5], Michiyo Higashi[6], Kenji Notohara[7], Shinichi Aishima[8,9], Nobuyuki Ohike[10], Takuma Tajiri[11], Hiroshi Yamaguchi[12], Yuki Fukumura[13], Motohiro Kojima[14], Kenichi Hirabayashi[15], Yoshihiro Hamada[16], Tomoko Norose[10], Keita Kai[9], Yuko Omori[17], Aoi Sukeda[18], Hirotsugu Noguchi[6], Kaori Uchino[7], Junya Itakura[7], Yoshinobu Okabe[19], Yuichi Yamada[5], Jun Akiba[2], Fahdi Kanavati[3], Yoshinao Oda[5], Toru Furukawa[17] & Hirohisa Yano[1]

1. Department of Pathology, Kurume University School of Medicine, Kurume, 830-0011 Japan
2. Department of Diagnostic Pathology, Kurume University Hospital, Kurume, 830-0011 Japan
3. Medmain Research, Medmain Inc., Fukuoka, 810-0042 Japan
4. Department of Pathology, Jichi Medical University, Shimotsuke, 329-0498, Tochigi
5. Department of Anatomic Pathology, Graduate School of Medical Sciences, Kyushu University, Fukuoka 812-8582 Japan
6. Department of Pathology, Research Field in Medicine and Health Sciences, Medical and Dental Sciences Area, Research and Education Assembly, Kagoshima University, Sakuragoaka, 890-8544, Japan
7. Department of Anatomic Pathology, Kurashiki Central Hospital, Kurashiki, 710-8602, Japan
8. Department of Pathology and Microbiology, Saga University, Saga, 849-8501, Japan
9. Department of Pathology, Saga University Hospital, Saga, 849-8501, Japan
10. Department of Pathology, Shizuoka Cancer Center, Shizuoka, 411-8777, Japan
11. Department of Pathology, Tokai University Hachioji Hospital, Tokyo, 192-0032, Japan
12. Department of Pathology, Saitama Medical University, Saitama, 350-0495, Japan
13. Department of Human Pathology, School of Medicine, Juntendo University, Tokyo, 113-8421, Japan

14. Division of Pathology, Exploratory Oncology Research & Clinical Trial Center, National Cancer Center, Kashiwa, 277-8577, Japan
15. Department of Pathology, Tokai University School of Medicine, Isehara, 259–1193, Japan
16. Department of Pathology, Fukuoka University Faculty of Medicine, Fukuoka University, Fukuoka, 814-0180, Japan
17. Department of Investigative Pathology, Tohoku University Graduate School of Medicine, Sendai, 980-8575 Japan
18. Department of Anatomic Pathology, Tokyo Medical University, Tokyo, 160-0023, Japan.
19. Division of Gastroenterology, Department of Medicine, Kurume University School of Medicine, Kurume, 830-0011 Japan

* These authors contributed equally to all experimental aspects of this work.

* **Corresponding authors:** Yoshiki Naito (nyoshiki@med.kurume-u.ac.jp) & Masayuki Tsuneki (tsuneki@medmain.com)

Supplementary Figure 1

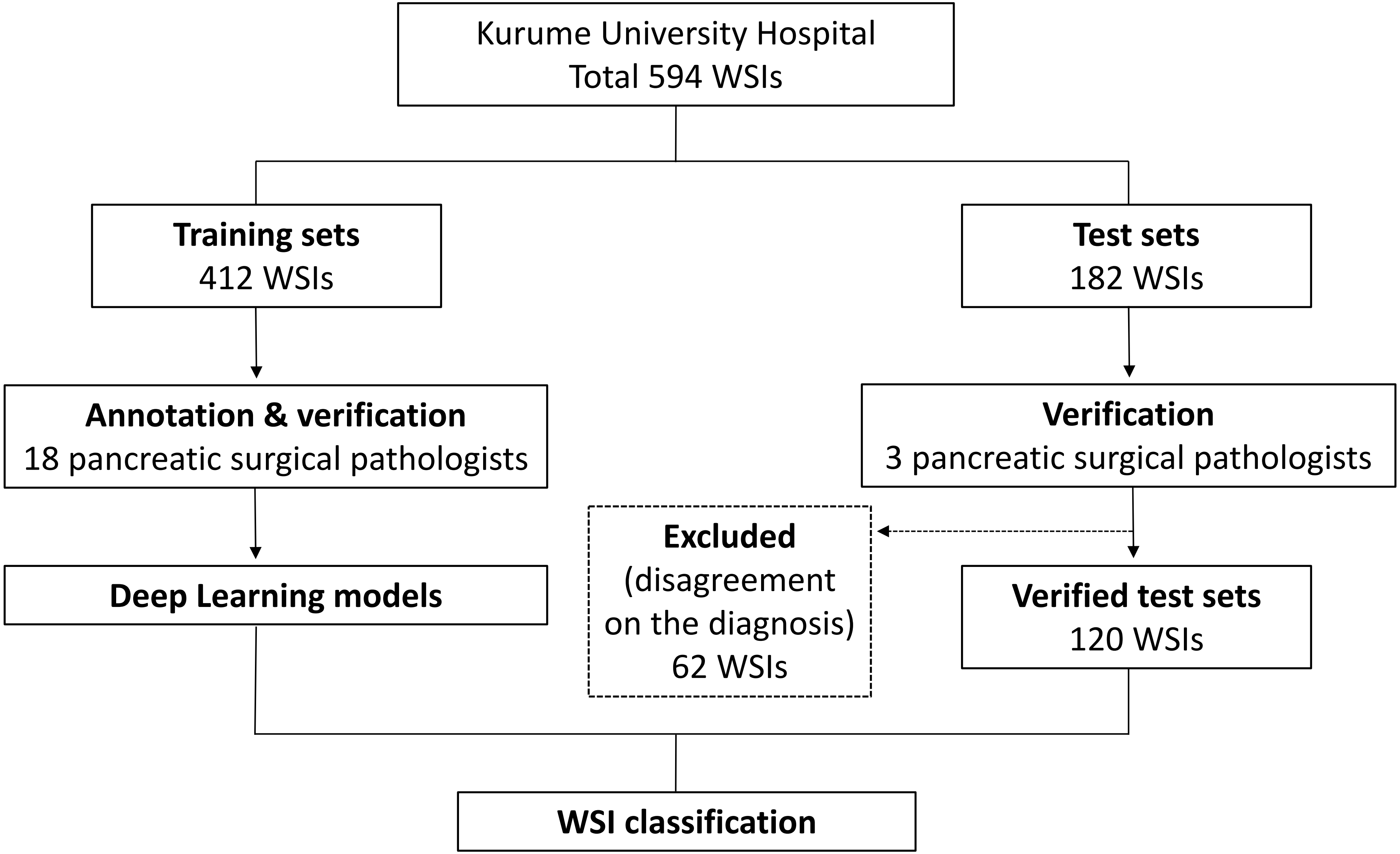

Supplementary Figure 2

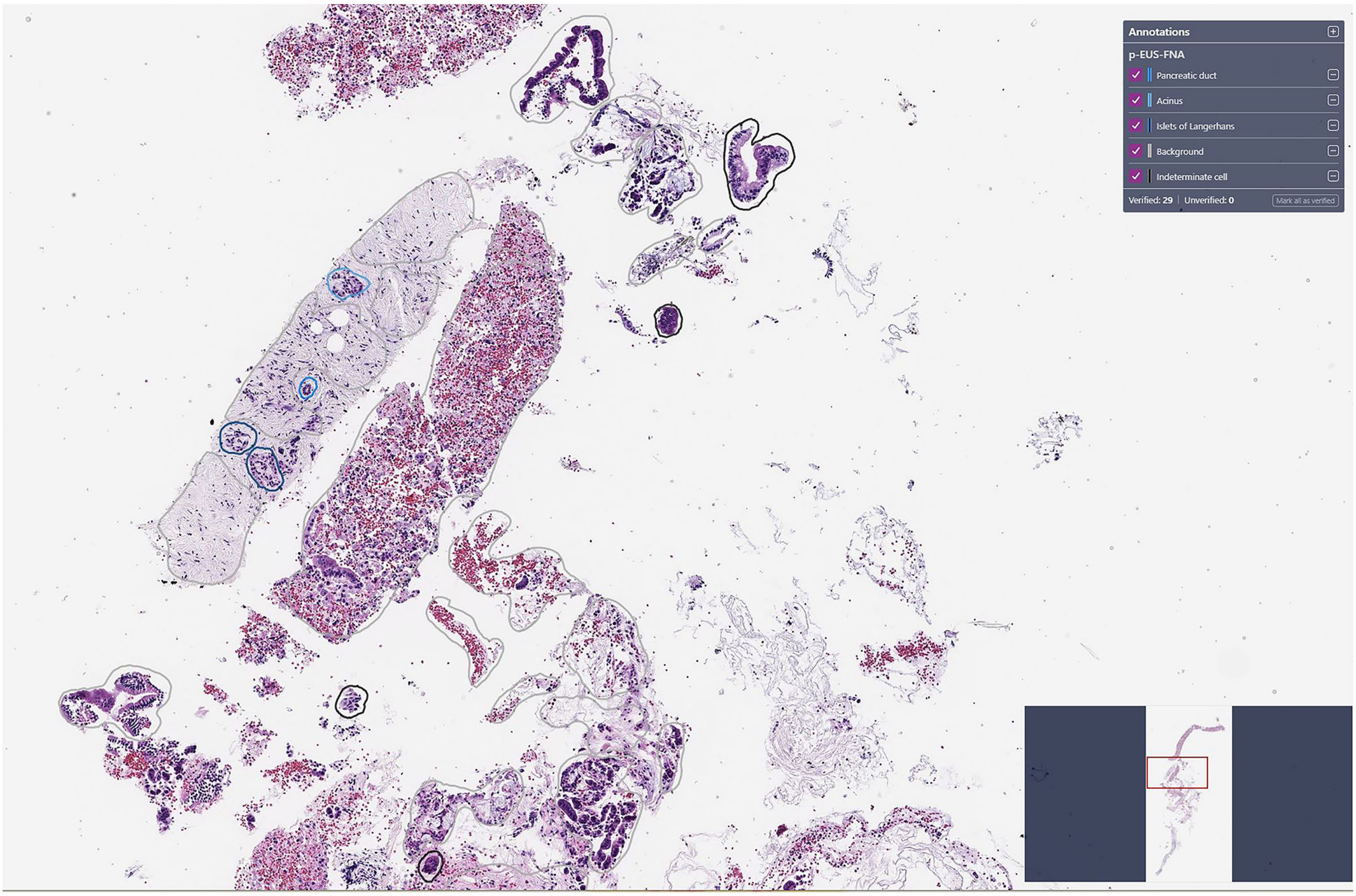

Supplementary Table 1. The results of pathological diagnoses of three pathologists on 182 WSIs				
**Case No.**	**Clinical diagnosis**	**Pathologist-1**	**Pathologist-2**	**Pathologist-3**
PAN-001	Autoimmuno pancreatitis	nonADC	nonADC	nonADC
PAN-002	Pancreatic cancer	ADC	ADC	ADC
PAN-003	Pancreatic cancer	ADC	ADC	ADC
PAN-004	Pancreatic cancer	ADC	ADC	ADC
PAN-005	Autoimmuno pancreatitis	nonADC	nonADC	nonADC
PAN-006	Pancreatic cancer	nonADC	indeterminate	indeterminate
PAN-007	Pancreatic cancer	nonADC	nonADC	nonADC
PAN-008	Pancreatic cancer	nonADC	nonADC	nonADC
PAN-009	Pancreatic cancer	nonADC	nonADC	nonADC
PAN-010	Autoimmuno pancreatitis	nonADC	nonADC	nonADC
PAN-011	Pancreatic cancer	nonADC	nonADC	nonADC
PAN-012	Pancreatic cancer	nonADC	nonADC	nonADC
PAN-013	Pancreatitis	nonADC	nonADC	nonADC
PAN-014	Pancreatic cancer	indeterminate	ADC	indeterminate
PAN-015	Autoimmuno pancreatitis	nonADC	nonADC	nonADC
PAN-016	Autoimmuno pancreatitis	nonADC	nonADC	nonADC
PAN-017	Pancreatic cancer	indeterminate	ADC	nonADC
PAN-018	Pancreatic cancer	ADC	ADC	ADC
PAN-019	Pancreatic cancer	ADC	ADC	ADC
PAN-020	Pancreatic cancer	ADC	ADC	ADC
PAN-021	Pancreatic cancer	nonADC	indeterminate	indeterminate
PAN-022	Pancreatic cancer	indeterminate	indeterminate	indeterminate
PAN-023	Autoimmuno pancreatitis	nonADC	nonADC	nonADC
PAN-024	Pancreatic cancer	ADC	ADC	ADC
PAN-025	Pancreatic cancer	ADC	ADC	ADC
PAN-026	Autoimmuno pancreatitis	nonADC	nonADC	nonADC
PAN-027	Neuroendocrine tumor	nonADC	nonADC	nonADC
PAN-028	Autoimmuno pancreatitis	nonADC	nonADC	nonADC
PAN-029	Autoimmuno pancreatitis	nonADC	nonADC	nonADC
PAN-030	Pancreatic cancer	ADC	ADC	ADC
PAN-031	Pancreatic cancer	ADC	ADC	nonADC
PAN-032	Neuroendocrine carcinoma	indeterminate	indeterminate	indeterminate
PAN-033	Neuroendocrine carcinoma	indeterminate	indeterminate	indeterminate
PAN-034	Neuroendocrine carcinoma	indeterminate	indeterminate	indeterminate
PAN-035	Pancreatic cancer	indeterminate	ADC	ADC
PAN-036	Pancreatic cancer	ADC	ADC	ADC
PAN-037	Pancreatic cancer	ADC	ADC	ADC
PAN-038	Pancreatic cancer	ADC	ADC	ADC
PAN-039	Pancreatic cancer	indeterminate	ADC	indeterminate
PAN-040	Pancreatic cancer	indeterminate	ADC	indeterminate
PAN-041	Pancreatic cancer	indeterminate	ADC	indeterminate
PAN-042	Pancreatic cancer	ADC	ADC	ADC
PAN-043	Pancreatic cancer	ADC	ADC	ADC
PAN-044	Pancreatic cancer	ADC	indeterminate	ADC
PAN-045	Pancreatic cancer	ADC	ADC	ADC
PAN-046	Autoimmuno pancreatitis	nonADC	nonADC	nonADC
PAN-047	Autoimmuno pancreatitis	nonADC	nonADC	nonADC
PAN-048	Pancreatic cancer	nonADC	nonADC	nonADC
PAN-049	Pancreatic cancer	ADC	ADC	ADC
PAN-050	Pancreatic cancer	ADC	ADC	ADC
PAN-051	Pancreatic cancer	ADC	ADC	ADC
PAN-052	Pancreatic cancer	ADC	ADC	ADC
PAN-053	Pancreatic cancer	ADC	ADC	ADC
PAN-054	Pancreatic cancer	indeterminate	ADC	ADC
PAN-055	Pancreatic cancer	ADC	ADC	ADC
PAN-056	Pancreatic cancer	ADC	ADC	ADC
PAN-057	Pancreatic cancer	ADC	ADC	ADC
PAN-058	Pancreatic cancer	ADC	ADC	ADC
PAN-059	Pancreatic cancer	ADC	ADC	ADC
PAN-060	Pancreatic cancer	indeterminate	indeterminate	ADC
PAN-061	Pancreatic cancer	ADC	ADC	ADC
PAN-062	Pancreatic cancer	ADC	ADC	ADC

PAN-063	Pancreatic cancer	nonADC	nonADC	nonADC
PAN-064	Pancreatic cancer	nonADC	nonADC	nonADC
PAN-065	Pancreatic cancer	nonADC	indeterminate	nonADC
PAN-066	Pancreatic cancer	ADC	ADC	ADC
PAN-067	Pancreatic cancer	indeterminate	ADC	nonADC
PAN-068	Pancreatic cancer	indeterminate	ADC	ADC
PAN-069	Pancreatic cancer	ADC	ADC	ADC
PAN-070	Autoimmuno pancreatitis	nonADC	nonADC	nonADC
PAN-071	Pancreatic cancer	ADC	indeterminate	ADC
PAN-072	Autoimmuno pancreatitis	nonADC	nonADC	nonADC
PAN-073	Autoimmuno pancreatitis	nonADC	nonADC	nonADC
PAN-074	Autoimmuno pancreatitis	nonADC	nonADC	nonADC
PAN-075	Autoimmuno pancreatitis	nonADC	nonADC	nonADC
PAN-076	Autoimmuno pancreatitis	nonADC	nonADC	nonADC
PAN-077	Pancreatic cancer	nonADC	nonADC	nonADC
PAN-078	Pancreatic cancer	indeterminate	ADC	nonADC
PAN-079	Pancreatic cancer	indeterminate	nonADC	nonADC
PAN-080	Pancreatic cancer	ADC	ADC	ADC
PAN-081	Pancreatic cancer	indeterminate	indeterminate	ADC
PAN-082	Pancreatic cancer	ADC	ADC	ADC
PAN-083	Pancreatic cancer	ADC	ADC	ADC
PAN-084	Pancreatic cancer	ADC	ADC	ADC
PAN-085	Pancreatic cancer	ADC	ADC	ADC
PAN-086	Pancreatic cancer	indeterminate	indeterminate	nonADC
PAN-087	Pancreatic cancer	ADC	nonADC	indeterminate
PAN-088	Pancreatic cancer	ADC	nonADC	indeterminate
PAN-089	Pancreatic cancer	ADC	ADC	ADC
PAN-090	Autoimmuno pancreatitis	nonADC	nonADC	nonADC
PAN-091	Autoimmuno pancreatitis	nonADC	nonADC	nonADC
PAN-092	Pancreatic cancer	ADC	ADC	ADC
PAN-093	Pancreatic cancer	ADC	ADC	ADC
PAN-094	Pancreatic cancer	ADC	ADC	ADC
PAN-095	Pancreatic cancer	ADC	ADC	ADC
PAN-096	Pancreatic cancer	ADC	ADC	ADC
PAN-097	Pancreatic cancer	ADC	ADC	ADC
PAN-098	Pancreatic cancer	nonADC	indeterminate	nonADC
PAN-099	Pancreatic cancer	ADC	ADC	ADC
PAN-100	Pancreatic cancer	ADC	indeterminate	indeterminate
PAN-101	Neuroendocrine tumor	indeterminate	indeterminate	indeterminate
PAN-102	Neuroendocrine tumor	indeterminate	indeterminate	indeterminate
PAN-103	Neuroendocrine tumor	indeterminate	indeterminate	indeterminate
PAN-104	Neuroendocrine tumor	indeterminate	indeterminate	indeterminate
PAN-105	Neuroendocrine tumor	indeterminate	indeterminate	indeterminate
PAN-106	Autoimmuno pancreatitis	nonADC	nonADC	nonADC
PAN-107	Autoimmuno pancreatitis	nonADC	nonADC	nonADC
PAN-108	Autoimmuno pancreatitis	nonADC	nonADC	nonADC
PAN-109	Autoimmuno pancreatitis	nonADC	nonADC	nonADC
PAN-110	Pancreatic cancer	ADC	ADC	ADC
PAN-111	Pancreatic cancer	ADC	ADC	ADC
PAN-112	Pancreatic cancer	ADC	ADC	ADC
PAN-113	Pancreatic cancer	ADC	ADC	ADC
PAN-114	Pancreatic cancer	indeterminate	nonADC	nonADC
PAN-115	Neuroendocrine tumor	indeterminate	indeterminate	indeterminate
PAN-116	Pancreatic cancer	ADC	ADC	ADC
PAN-117	Pancreatic cancer	indeterminate	ADC	indeterminate
PAN-118	Pancreatic cancer	indeterminate	ADC	ADC
PAN-119	Pancreatic cancer	ADC	ADC	ADC
PAN-120	Pancreatic cancer	indeterminate	ADC	ADC
PAN-121	Pancreatic cancer	nonADC	indeterminate	ADC
PAN-122	Pancreatic cancer	indeterminate	ADC	ADC
PAN-123	Pancreatic cancer	ADC	ADC	ADC
PAN-124	Pancreatic cancer	nonADC	indeterminate	nonADC
PAN-125	Pancreatic cancer	ADC	indeterminate	indeterminate
PAN-126	Pancreatic cancer	ADC	ADC	ADC
PAN-127	Pancreatic cancer	nonADC	ADC	indeterminate

PAN-128	Pancreatic cancer	indeterminate	ADC	indeterminate
PAN-129	Pancreatic cancer	indeterminate	ADC	indeterminate
PAN-130	Pancreatic cancer	ADC	ADC	ADC
PAN-131	Pancreatic cancer	ADC	ADC	ADC
PAN-132	Pancreatic cancer	ADC	ADC	ADC
PAN-133	Pancreatic cancer	ADC	ADC	ADC
PAN-134	Pancreatic cancer	indeterminate	ADC	ADC
PAN-135	Pancreatic cancer	ADC	ADC	ADC
PAN-136	Pancreatic cancer	indeterminate	ADC	ADC
PAN-137	Pancreatic cancer	ADC	ADC	ADC
PAN-138	Pancreatic cancer	ADC	ADC	ADC
PAN-139	Pancreatic cancer	ADC	ADC	ADC
PAN-140	Pancreatic cancer	ADC	ADC	ADC
PAN-141	Pancreatic cancer	ADC	ADC	ADC
PAN-142	Pancreatic cancer	indeterminate	ADC	indeterminate
PAN-143	Pancreatic cancer	indeterminate	ADC	ADC
PAN-144	Pancreatic cancer	ADC	ADC	ADC
PAN-145	Pancreatic cancer	ADC	ADC	ADC
PAN-146	Neuroendocrine tumor	indeterminate	indeterminate	indeterminate
PAN-147	Pancreatic cancer	ADC	nonADC	ADC
PAN-148	Pancreatic cancer	ADC	ADC	ADC
PAN-149	Pancreatic cancer	ADC	ADC	ADC
PAN-150	Pancreatic cancer	indeterminate	ADC	ADC
PAN-151	Pancreatic cancer	ADC	ADC	ADC
PAN-152	Pancreatic cancer	ADC	ADC	ADC
PAN-153	Pancreatic cancer	indeterminate	indeterminate	indeterminate
PAN-154	Pancreatic cancer	ADC	ADC	ADC
PAN-155	Pancreatic cancer	ADC	ADC	ADC
PAN-156	Neuroendocrine tumor	indeterminate	indeterminate	indeterminate
PAN-157	Pancreatic cancer	ADC	ADC	ADC
PAN-158	Pancreatic cancer	ADC	ADC	ADC
PAN-159	Pancreatic cancer	ADC	ADC	ADC
PAN-160	Pancreatic cancer	nonADC	indeterminate	indeterminate
PAN-161	Pancreatic cancer	nonADC	nonADC	nonADC
PAN-162	Pancreatic cancer	nonADC	indeterminate	nonADC
PAN-163	Pancreatic cancer	ADC	ADC	ADC
PAN-164	Pancreatic cancer	ADC	ADC	ADC
PAN-165	Pancreatic cancer	ADC	ADC	ADC
PAN-166	Pancreatic cancer	ADC	ADC	ADC
PAN-167	Pancreatic cancer	ADC	ADC	ADC
PAN-168	Pancreatic cancer	ADC	ADC	ADC
PAN-169	Pancreatic cancer	ADC	ADC	ADC
PAN-170	Pancreatic cancer	ADC	ADC	ADC
PAN-171	Pancreatic cancer	ADC	ADC	ADC
PAN-172	Pancreatic cancer	ADC	ADC	ADC
PAN-173	Pancreatic cancer	ADC	ADC	ADC
PAN-174	Pancreatic cancer	nonADC	ADC	indeterminate
PAN-175	Pancreatic cancer	indeterminate	ADC	indeterminate
PAN-176	Pancreatic cancer	ADC	ADC	ADC
PAN-177	Pancreatic cancer	indeterminate	ADC	nonADC
PAN-178	Pancreatic cancer	indeterminate	ADC	indeterminate
PAN-179	Pancreatic cancer	indeterminate	indeterminate	nonADC
PAN-180	Pancreatic cancer	ADC	ADC	ADC
PAN-181	Pancreatic cancer	ADC	ADC	ADC
PAN-182	Pancreatic cancer	ADC	ADC	ADC
